# Hepatic immunometabolic signatures associated with the heterophil-to-lymphocyte ratio reveal sex-dependent patterns in yellow-legged ma chickens

**DOI:** 10.1016/j.psj.2026.107519

**Published:** 2026-07-29

**Authors:** Shihao Chen, Qiangzhou Wang, Xuan Huang, Qingqing Cai, Xiaoxian Ru, Pengfei Chu, Hao Bai, Guobin Chang

**Affiliations:** a College of Animal Science and Technology, Yangzhou University, 48 East Wenhui Road, Yangzhou, Jiangsu, China; b College of Veterinary Medicine, Yangzhou University, Yangzhou, 225009, Jiangsu, China

**Keywords:** Heterophil-to-lymphocyte ratio, Sex-dependent patterns, Immunometabolism, Yellow-legged Ma chicken

## Abstract

The peripheral blood heterophil-to-lymphocyte ratio (H/L ratio) is widely used as an immunophenotypic indicator in poultry, but its tissue-level molecular basis remains unclear. In this study, individuals with extreme high and low H/L ratios were selected from 113 Yellow-legged Ma chickens, and sex stratification was incorporated. Liver and spleen histology, serum biochemical and oxidative stress indices, transcriptomic data, and metabolomic data were integrated to characterize H/L-associated hepatic immunometabolic features. The results showed no significant differences between the high- and low-H/L groups in liver and spleen histological morphology, serum ALT and AST activities, or oxidative stress indicators, suggesting that H/L variation indicating that H/L variation was not associated with overt histopathological changes, liver injury, or oxidative imbalance under the conditions examined. Multi-omics analyses revealed clear sex-specific molecular patterns. In hens, low-H/L individuals showed higher expression of several immune-related genes, and the differential features mainly involved T cell receptor signaling, cytokine–cytokine receptor interaction, Th cell differentiation, arginine biosynthesis, α-linolenic acid metabolism, and pyrimidine metabolism. In roosters, H/L-associated differences mainly involved nuclease activity, chemokine signaling, JAK-STAT signaling, Toll-like receptor signaling, histidine metabolism, and glutathione metabolism. Integrative analysis identified CCR2 as a potential candidate node that may participate in H/L-related hepatic immunometabolic remodeling. Overall, the H/L ratio is associated with the basal hepatic immunometabolic status of Yellow-legged Ma chickens in a sex-specific manner, providing a molecular basis for further evaluating its application in poultry immune phenotyping and disease-resistance breeding.

## Introduction

The peripheral blood heterophil-to-lymphocyte ratio (H/L ratio) is widely regarded as an indicator of immune status and has been extensively used in poultry disease-resistance assessment (Cotter, 2015; Scanes, 2016). Previous studies have suggested that a lower H/L ratio is generally associated with improved health status and enhanced resistance to pathogens (Thiam et al., 2022b; Wang et al., 2020). However, the biological mechanisms underlying inter-individual variation in the H/L ratio remain incompletely understood, particularly with respect to tissue-level immune and metabolic regulation.

The liver is a central metabolic organ and an important immunometabolic hub that participates in inflammatory regulation, energy allocation, oxidative stress responses, and the maintenance of immune homeostasis (Zaefarian et al., 2019). These functions make the liver a critical tissue for sustaining basal immune readiness and coordinating systemic physiological responses (Kawashima et al., 2024). Therefore, whether differences in the peripheral H/L ratio are associated with distinct hepatic physiological states and immunometabolic capacities warrants further investigation. In particular, it remains unclear whether chickens with a low H/L ratio exhibit a hepatic molecular profile that is more conducive to immune homeostasis, redox balance, and immune response priming.

Biological sex is another important factor influencing immune and metabolic regulation (Klein and Flanagan, 2016). Increasing evidence indicates that sex, through the combined effects of genetic background, sex hormones, and environmental interactions, contributes to immune sexual dimorphism across tissues (Dunn et al., 2024; Kasarinaite et al., 2023; Sharma et al., 2025). However, sex has often not been sufficiently incorporated into analytical frameworks in studies of H/L ratio-related phenotypes (Wang et al., 2023), which may mask sex-specific molecular features and potential biomarkers. Therefore, incorporating sex into the analysis of H/L-associated hepatic molecular characteristics may improve the biological interpretation of the H/L ratio and enhance its potential utility in poultry health assessment and disease-resistance breeding.

In this study, Yellow-legged Ma chickens were used as the experimental model. Individuals with high and low peripheral blood H/L ratios were selected, and sex was included as a key analytical factor. By integrating liver histology, serum biochemical and oxidative stress indices, transcriptomics, and metabolomics, we systematically characterized hepatic immunometabolic features associated with different H/L states. This study reveals H/L ratio-associated and sex-specific hepatic molecular landscapes, providing biological insight into the use of the H/L ratio as an immune phenotype marker and offering a theoretical basis for disease-resistance breeding in poultry.

## Materials and methods

### Animals, experimental design and grouping

Yellow-legged Ma chickens were obtained from Yangzhou Lihua Poultry Breeding Co., Ltd. A total of 113 birds were reared under identical environmental conditions, with feed and water provided ad libitum. All birds were confirmed to be negative for major pathogens, and no clinical signs were observed throughout the study. On day 56, peripheral blood was collected from 113 chickens to calculate the H/L ratio. Based on the distribution of H/L ratios, individuals with extreme values were selected, including 11 chickens with high H/L ratios, comprising 5 males and 6 females, and 10 chickens with low H/L ratios, comprising 4 males and 6 females. These chickens were further classified by sex into four subgroups.

### Determination of the H/L ratio

Blood was collected from the wing vein, and blood smears were prepared and stained with Wright’s stain. Leukocyte morphology was examined under a light microscope at 1,000× magnification (10 × 100). Heterophils and lymphocytes were differentially counted, with 100 leukocytes counted per smear. Two blood smears were examined for each sample, and the H/L ratio was calculated as the number of heterophils divided by the number of lymphocytes.

### Sample collection and processing

The chickens were euthanized after anesthesia, and liver tissues were immediately collected following dissection. A portion of the liver and spleen tissues were fixed in 4% paraformaldehyde for histological observation. The remaining liver samples were quickly frozen in liquid nitrogen and transferred to −80°C for transcriptomic and metabolomic analyses. Simultaneously, blood samples were collected and allowed to stand at room temperature. The serum was then separated by centrifugation at 3000 rpm for 10–15 min and stored at 4°C for subsequent use.

### Histological analysis

Fixed liver and spleen tissues were processed through routine dehydration, clearing, and paraffin embedding, followed by sectioning. The sections were stained with hematoxylin and eosin (H&E) to observe histomorphological changes under a light microscope. For each sample, three fields of view were randomly selected for image acquisition.

### Determination of serum biochemical and oxidative stress parameters

Serum alanine aminotransferase (ALT) and aspartate aminotransferase (AST) activities were measured by AiFang Biotechnology Co., Ltd. (Changsha, Hunan, China). Total antioxidant capacity (T-AOC), superoxide dismutase (SOD), glutathione peroxidase (GSH-Px), and malondialdehyde (MDA) levels were determined using commercial assay kits (Puxi Biotech, Shanghai, China). Five replicate wells were used for each parameter, and the final results were calculated according to the manufacturers’ instructions.

### Transcriptomic sequencing and bioinformatics analysis

All 21 liver samples were subjected to RNA-seq: FH (n = 6), FL (n = 6), MH (n = 5), and ML (n = 4), with each sample representing an independent biological replicate. Established protocols for transcriptomic sequencing and data processing were followed (Wang et al., 2025). Total RNA was extracted from liver tissues using TRIzol reagent (Vazyme, Nanjing, China). Sequencing was performed on the Illumina NovaSeq 6000 platform by Gene Denovo (Guangzhou, China). Differentially expressed genes (DEGs) were identified based on the criteria of |log2FoldChange| > 1 along with either p-value < 0.01 or FDR < 0.05. Comparisons were conducted between the overall high and low H/L groups, as well as within the female and male subgroups separately. Gene Ontology (GO) and Kyoto Encyclopedia of Genes and Genomes (KEGG) annotation and enrichment analyses were performed using the OmicShare platform (https://www.omicsmart.com), with the top 15 terms selected for visualization based on their FDR values.

### Real-time quantitative PCR

Total RNA was extracted using TRIzol and reverse transcribed into cDNA using HiScript III RT SuperMix for qPCR (+gDNA wiper) (Vazyme, Nanjing, China). Real-time quantitative PCR (RT-qPCR) was performed using the ChamQ Universal SYBR qPCR Master Mix (Vazyme, Nanjing, China) on a Bio-Rad CFX Connect system. Gene sequences were obtained from the NCBI database, and primers were designed using Primer Premier 6.0. The primers were synthesized by Azenta (Genewiz, Suzhou), and the specific primer sequences are listed in Table 1.

**Table 1:** List of primer sequences for RT-qPCR analysis.

Gene	Primer sequences (5′−3′)
*MSTRG.6421*	F: GGAGTTGACAATGATGAAGT
	R: GAGAGTATCGTAGATGGATTC
*ITIH5*	F: GGAATCTGGTGTAACTGTGA
	R: TGGCTTATTGCTGAGGATAG
*IFNLR1*	F: CTACCATCATCATCATCAGAAG
	R: ATCCACCAACCACAGAGA
*CCR2*	F: GGCAGCACTCCTACATTC
	R: AAGGTGTCCAGAAGATGAAG
*MAOA*	F: AAGGAGGAGAGGAAGAAGA
	R: TGAATACATTATGCCAGGTG
*MXRA7*	F: GAGGAAGAGGAGGAGGAA
	R: CATCATTGACTTGTACTGGT
*β-ACTIN*	F: GAGAAATTGTGCGTGACATCA
	R: CCTGAACCTCTCATTGCCA

### Metabolomic analysis

The same 21 liver samples were subjected to untargeted metabolomic profiling: FH (n = 6), FL (n = 6), MH (n = 5), and ML (n = 4), with each sample representing an independent biological replicate. Metabolomic profiling, including mass spectrometry and bioinformatics analyses, was performed in collaboration with Gene Denovo Biotechnology Co., Ltd. (Guangzhou, China).Samples from hens and roosterswere analyzed independently in both positive and negative ion modes. The raw mass spectrometry data were processed through peak extraction, alignment, normalization, and denoising to obtain the metabolite feature matrix. Differentially abundant metabolites were identified using OPLS-DA combined with univariate statistics, based on the criteria of VIP > 1 and *P* < 0.05. Topology analyses were performed on the identified differential metabolites.

### Integrated analysis of transcriptome and metabolome

The O2PLS method was used to evaluate the synergistic relationship between genes and metabolites, and a gene-metabolite correlation network was constructed based on Pearson correlation analysis. Gene-metabolite pairs with an absolute correlation coefficient greater than 0.5 were selected for visualization. Furthermore, key candidate regulatory nodes were identified by integrating shared enriched pathways.

### Statistical analysis

All data are presented as mean ± standard deviation (mean ± SD). Comparisons between two groups were performed using independent sample t-tests, while multiple group comparisons were conducted using one-way ANOVA followed by Tukey's multiple comparison test. Statistical analyses were performed using GraphPad Prism 9.5. *P* < 0.05 was considered statistically significant.

## Results

### Characterization of H/L ratio groups and hepatic basal physiological state

**Fig. 1:**
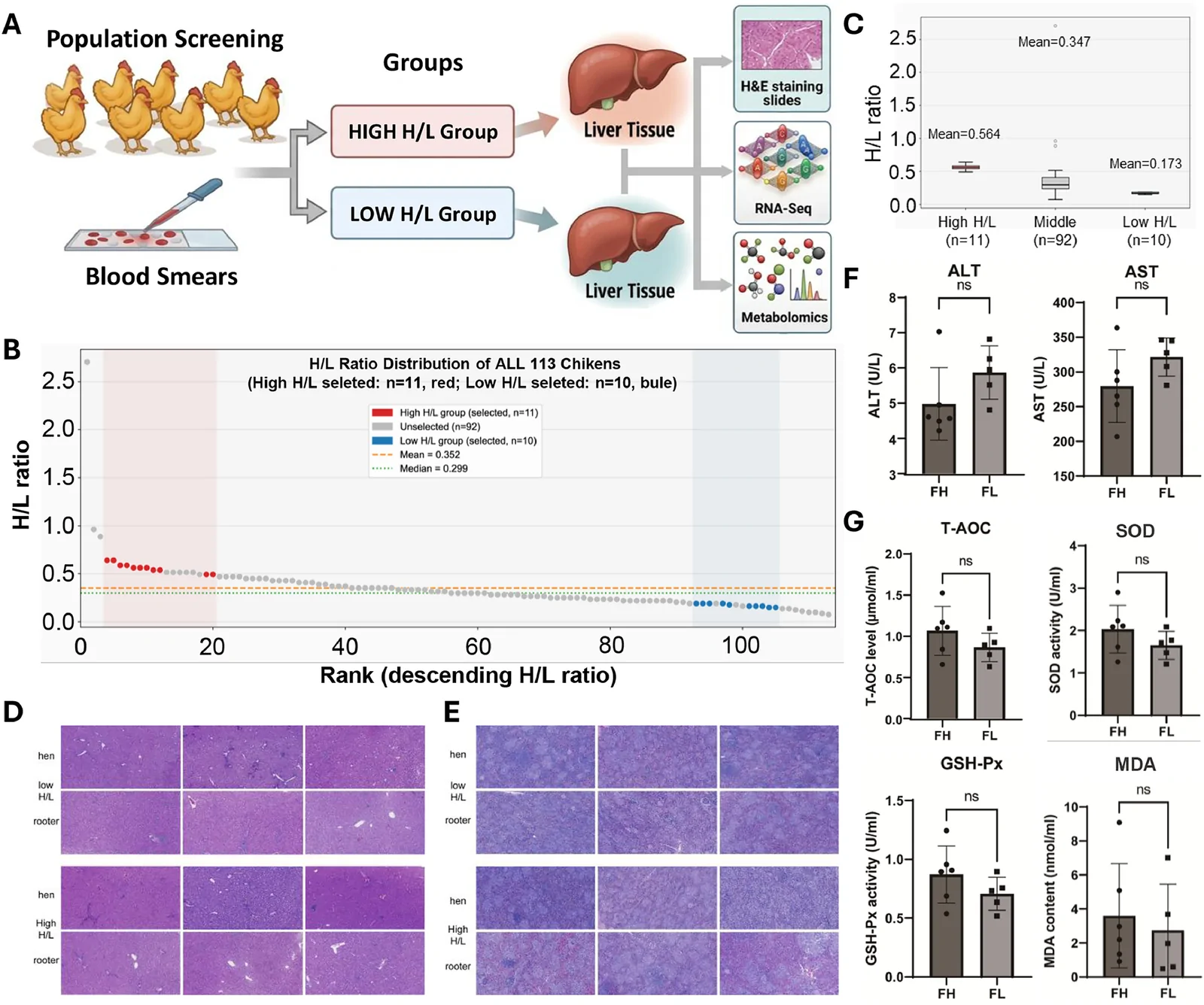
Screening of individuals with divergent H/L ratios and evaluation of baseline physiological status. (A) Schematic overview of the workflow of this study. (B) Distribution of H/L ratios in the whole population (n = 113), showing selection of the high-H/L and low-H/L extreme individuals. (C) Boxplot showing H/L ratio distributions of the high-H/L, middle and low-H/L populations. (D–E) Representative histological images of liver and spleen tissues from high- and low-H/L hens and roosters. (F) Serum ALT and AST activities in the female high-H/L (FH) and female low-H/L (FL) groups. (G) Oxidative stress-related indices, including T-AOC, SOD, GSH-Px and MDA, in FH and FL groups. Data are presented as mean ± SD; ns, not significant.

Based on the H/L ratio determined from peripheral blood smears, we performed a population screening of 113 chickens. Eleven individuals from the high end and ten from the low end of the H/L ratio distribution were selected and defined as the high H/L group (H group, n = 11) and the low H/L group (L group, n = 10) (Fig. 1A, B). The H/L ratios of the two groups were clearly separated, with an average ratio of 0.564 for the H group and 0.173 for the L group, compared to the overall population mean of 0.347 (Fig. 1C).

To assess the basal hepatic state of the individuals at the time of sampling, histological observations were performed on liver and spleen tissues. The results showed that the overall structure of the liver and spleen tissues in both H and L groups remained intact, with no obvious inflammatory infiltration, necrosis, or other significant pathological damage (Fig. 1D, E). Consistent with this, serum biochemical analysis revealed no significant differences in ALT and AST activities between the two groups (P > 0.05, Fig. 1F). Furthermore, oxidative stress-related indicators, including T-AOC, SOD, GSH-Px, and MDA, showed no significant changes between the groups (P > 0.05, Fig. 1G). These results indicate that the selected high and low H/L individuals showed no detectable evidence of overt liver injury or oxidative stress imbalance under the conditions examined.. Therefore, the differences in H/L ratio observed in this study were not associated with the overt pathological changes assessed here.

### Sex-dependent hepatic transcriptomic divergence

**Fig. 2:**
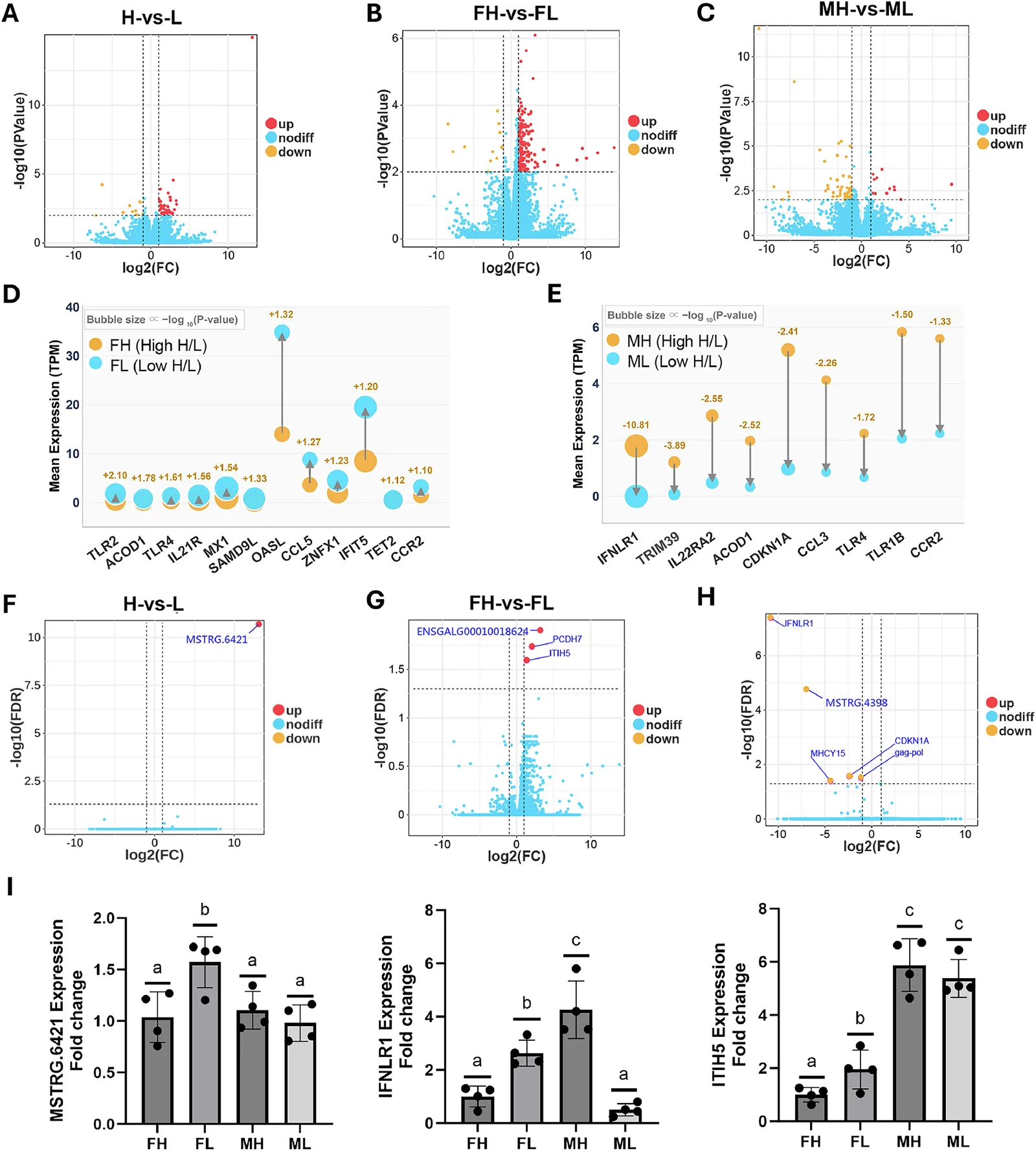
Sex-stratified transcriptomic landscapes associated with H/L ratio. (A–C) Volcano plots of differentially expressed genes (DEGs) in the overall comparison (A), the female high-H/L (FH) versus female low-H/L (FL) comparison (B), and the male high-H/L (MH) versus male low-H/L (ML) comparison (C). (D, E) Lollipop/bubble plots showing the mean expression levels (TPM) of representative DEGs in the female (FH vs FL, D) and male (MH vs ML, E) subgroup comparisons. Bubble size is proportional to −log10(P value), and the numbers indicate log2(fold change). (F–H) FDR-based volcano plots highlighting representative significant genes/transcripts in the overall comparison (F), the female subgroup comparison (FH vs FL, G), and the male subgroup comparison (MH vs ML, H). (I) RT-qPCR validation of selected candidate transcripts/genes, including MSTRG.6421, IFNLR1, and ITIH5, in the FH, FL, MH, and ML groups. Different lowercase letters indicate significant differences among groups (*P* < 0.05).

RNA-seq differential expression analysis revealed that in the overall comparison, a total of 50 DEGs were identified between the high and low H/L groups, with 38 genes upregulated and 12 genes downregulated in the low H/L group (Fig. 2A). In contrast, the differential expression profiles became more pronounced after sex stratification. In the female group comparison (FH vs. FL), 185 DEGs were detected, of which 172 were relatively higher in the low H/L group and only 13 were higher in the high H/L group (Fig. 2B). Representative DEGs included *TLR4, MX1, SAMD9L, OASL, IFIT5*, and *CCR2* (Fig. 2D). Most of these genes are associated with innate immunity, interferon responses, and inflammatory reactions, suggesting that the differences in the female low H/L group are primarily accompanied by an enhancement of immune-related transcriptional programs. In the male group comparison (MH vs. ML), 81 DEGs were identified, with 69 relatively higher in the high H/L group and 12 higher in the low H/L group (Fig. 2C). Representative DEGs included *IFNLR1, IL22RA2, CCL3, TLR4, IL7R*, and *CCR2* (Fig. 2E). Given that these DEGs were relatively higher in the high H/L group, this indicates that the high H/L group in males may possess stronger inflammatory/immune-related transcriptional signatures than the low H/L group.

Volcano plots adjusted by FDR further identified representative candidate genes or transcripts for each comparison: *MSTRG.6421* was representative in the overall H/L comparison (Fig. 2F); *IFNLR1, MHCY15*, and *CDKN1A* were identified as significant candidate genes in the hen groups (Fig. 2G); while *ITH5* and *PCDH7* were representative in the rooster groups (Fig. 2H). This suggests that H/L-related molecular features exhibit sex-specific transcriptional response patterns. RT-qPCR validation results generally supported the RNA-seq analysis (Fig. 2i). Taken together, the transcriptional responses associated with low H/L ratios are highly sex-dependent: hens tend to show widespread transcriptional activation, while roosters exhibit relatively conservative or inhibitory expression remodeling.

### Functional enrichment defines distinct H/L-associated pathways

**Fig. 3:**
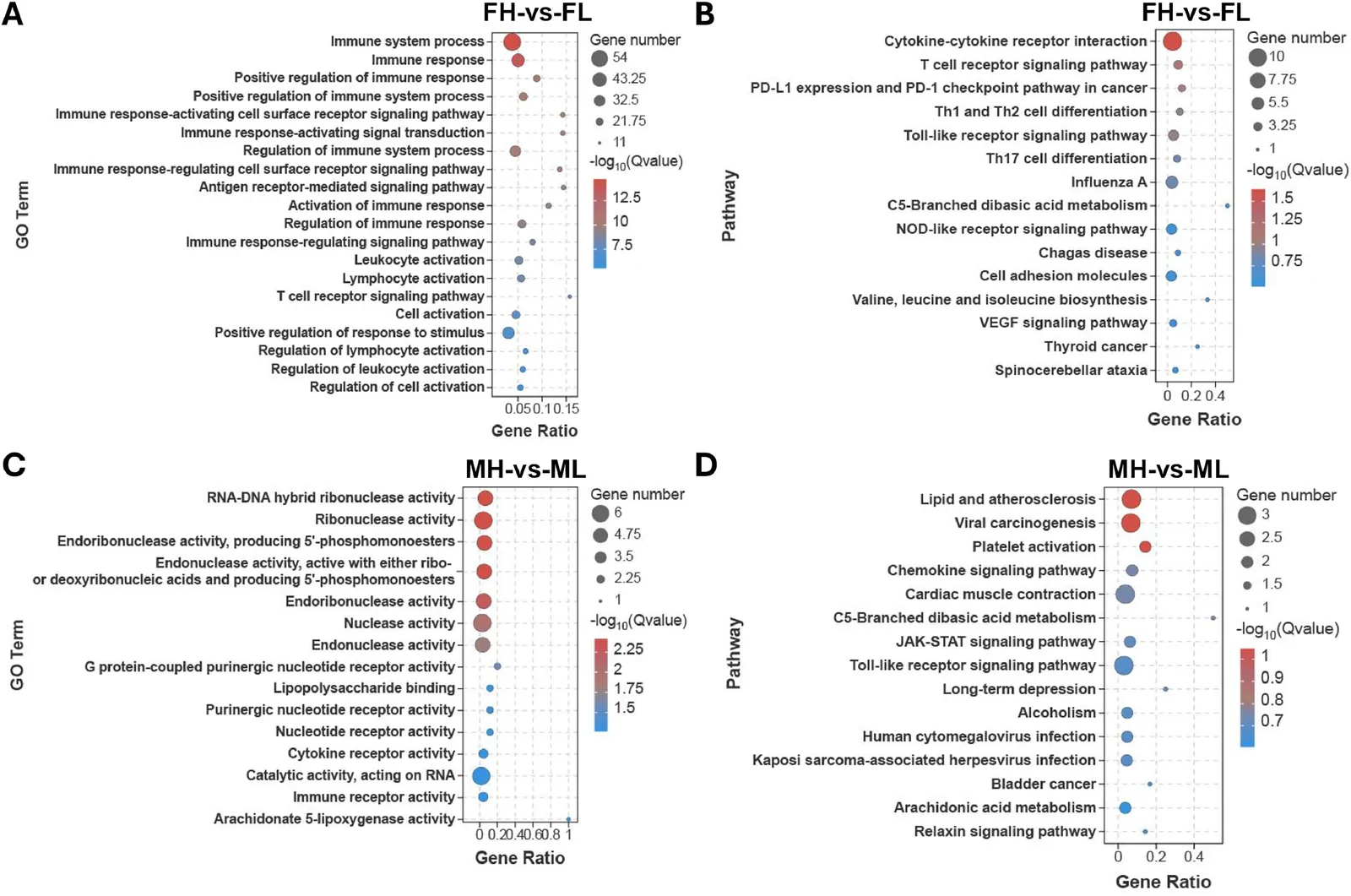
Functional enrichment of liver DEGs in hens and roosters. (A) GO enrichment analysis of DEGs identified in hens. (B) KEGG pathway enrichment analysis of DEGs identified in hens. (C) GO enrichment analysis of DEGs identified in roosters. (D) KEGG pathway enrichment analysis of DEGs identified in roosters. Dot size indicates gene number, and color intensity indicates enrichment significance.

GO and KEGG enrichment analyses of sex-stratified DEGs revealed distinct functional biases between hens and roosters. In hens, GO terms were primarily concentrated in immune system processes, immune response, activation of immune response signaling pathways, antigen receptor-mediated signaling pathways, leukocyte/lymphocyte activation, and T cell receptor signaling pathways (Fig. 3A). KEGG analysis further showed that these genes were significantly enriched in cytokine-cytokine receptor interaction, T cell receptor signaling pathway, PD-L1/PD-1 checkpoint pathway, Th1/Th2 cell differentiation, Th17 cell differentiation, and Toll-like receptor signaling pathway (Fig. 3B). These results indicate that transcriptomic changes associated with the H/L ratio in females mainly involve immune activation, lymphocyte response, and T cell-related regulatory networks.

In contrast, GO enrichment in the rooster group prominently featured RNA-DNA hybrid ribonuclease activity, ribonuclease activity, endonuclease activity, nuclease activity, and several receptor activity-related terms (Fig. 3C), suggesting that H/L-related transcriptional differences in roosters are more closely linked to nucleic acid processing and recognition. KEGG analysis showed that DEGs in males were mainly enriched in lipid and atherosclerosis, viral carcinogenesis, platelet activation, chemokine signaling pathway, JAK-STAT signaling pathway, and Toll-like receptor signaling pathway (Fig. 3D). Given that most DEGs in the MH-vs-ML comparison were upregulated in the high H/L group (Fig. 2E), these enriched pathways appear to be more active in the MH group. This suggests that high H/L roosters may possess more prominent transcriptional signatures related to inflammatory-receptor signaling and lipid metabolism.

### Sex-biased hepatic metabolomic remodeling

**Fig. 4:**
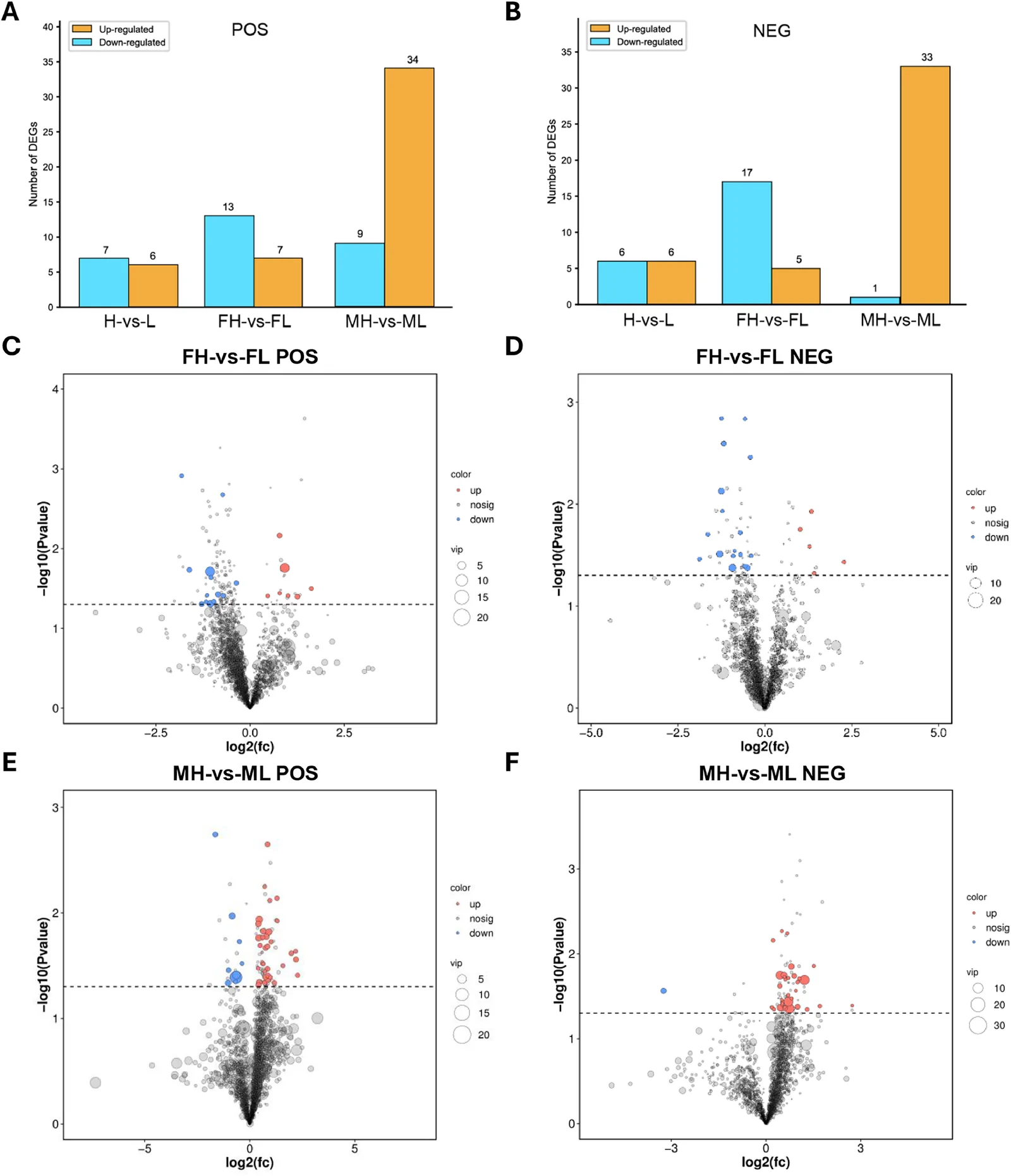
Sex-stratified differential metabolite profiles associated with H/L ratio. (A–B) Summary of increased and decreased -regulated differential metabolic features detected in different comparison sets under positive and negative ion modes. (C–F) Volcano plots showing differential metabolites in hens under positive ion mode, hens under negative ion mode, roosters under positive ion mode, and roosters under negative ion mode, respectively. Each dot represents one metabolic feature; red and blue indicate significantly increased and decreased metabolites, respectively, and grey indicates non-significant features.

Untargeted metabolomic analysis revealed that H/L ratio-related alterations in liver metabolism are also clearly sex-dependent. Based on volcano plot screening, 20 and 22 differential metabolites were identified in hens under positive and negative ion modes, respectively, totaling 42 metabolites. In roosters, 43 and 34 differential metabolites were identified, totaling 77 metabolites (Fig. 4A, B). Overall, the number of differential metabolites in roosters was significantly higher than in hens, suggesting that roosters undergo more extensive metabolic remodeling in the context of H/L ratio stratification.

The volcano plots showed that H/L ratio stratification was associated with differential metabolic signatures in hens under both positive and negative ion modes, although the total number of differential metabolites was relatively limited (Fig. 4C, D). In roosters, H/L ratio stratification was associated with a larger number of differential metabolites in both ion modes, with particularly pronounced changes observed in the positive ion mode (Fig. 4E, F). These results indicate that H/L ratio-related metabolic differences are present in both sexes but are more extensively reflected as hepatic metabolic reprogramming in roosters than in hens.

Integrating these findings with the aforementioned transcriptomic data reveals that low H/L hens exhibit stronger immune-related differences at the transcriptional level, while low H/L roosters show more significant metabolomic changes. This suggests that hens and roosters may employ different levels of regulatory strategies in their biological responses associated with the H/L ratio.

### H/L-associated metabolic pathway priorities differ between hens and roosters

**Fig. 5:**
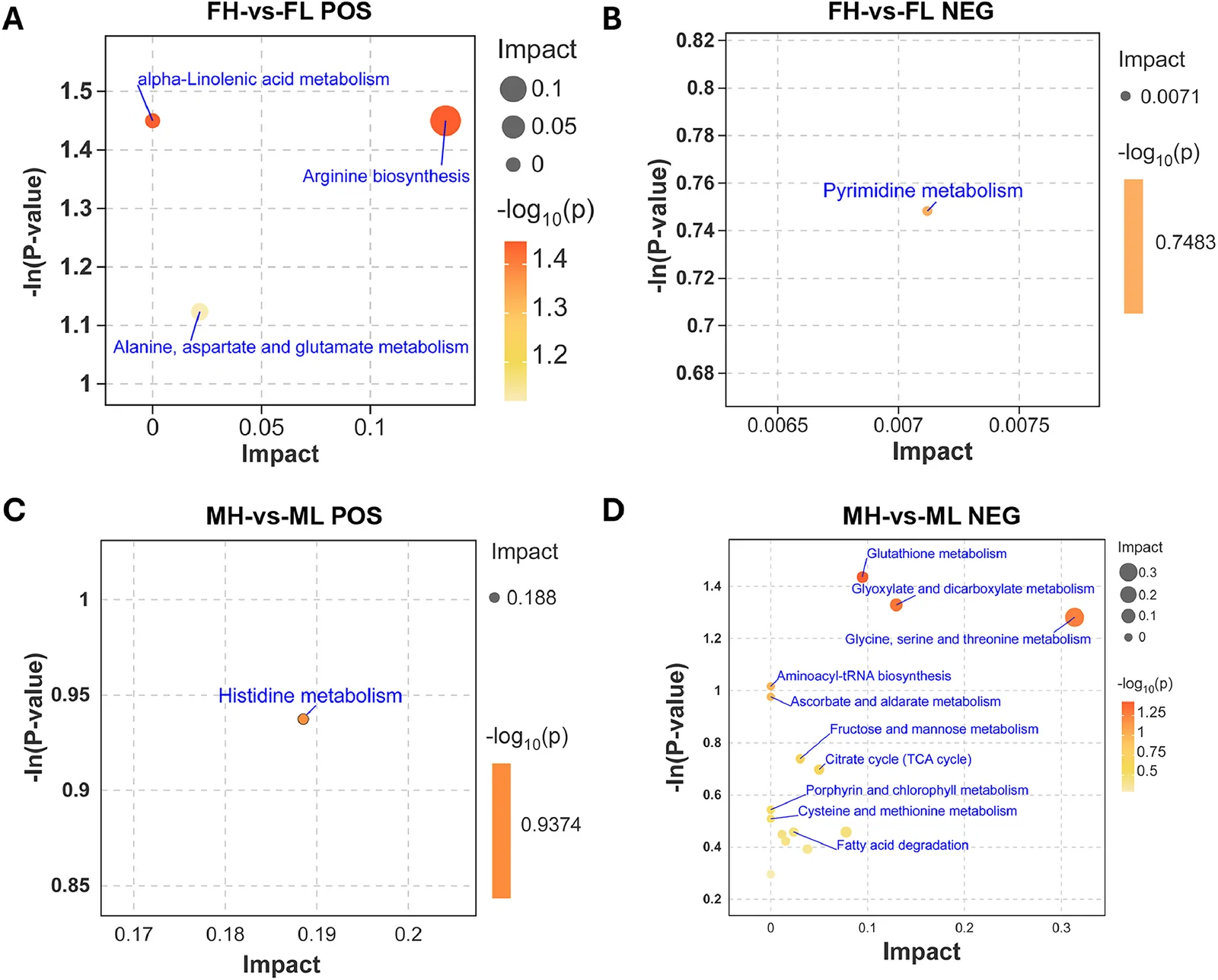
Pathway topology analysis of differential metabolites in hens and roosters. (A) Enriched metabolic pathways in hens under positive ion mode. (B) Enriched metabolic pathways in hens under negative ion mode. (C) Enriched metabolic pathways in roosters under positive ion mode. (D) Enriched metabolic pathways in roosters under negative ion mode. Bubble size represents pathway impact, and color indicates enrichment significance.

Pathway topology analysis further demonstrated that H/L ratio-related metabolic changes exhibit distinct pathway biases between the sexes. In the positive ion mode for hens, differential metabolites were primarily mapped to arginine biosynthesis, alpha-linolenic acid metabolism, and alanine, aspartate, and glutamate metabolism. Arginine biosynthesis showed a high pathway impact value, while alpha-linolenic acid metabolism exhibited relatively higher statistical significance (Fig. 5A). In the negative ion mode for hens, only the pyrimidine metabolism pathway was enriched (Fig. 5B), suggesting that H/L-related metabolic changes in hens are mainly concentrated in amino acid, lipid-related, and nucleotide metabolic processes.

In contrast, the positive ion mode for roosters was mainly enriched in the histidine metabolism pathway, which had a relatively high pathway impact value (Fig. 5C). The negative ion mode for roosters revealed significantly more enriched pathways, including glutathione metabolism, glyoxylate and dicarboxylate metabolism, glycine/serine/threonine metabolism, aminoacyl-tRNA biosynthesis, ascorbate and aldarate metabolism, fructose and mannose metabolism, the citrate cycle (TCA cycle), cysteine and methionine metabolism, and fatty acid degradation (Fig. 5D). These results indicate that H/L-related metabolic remodeling in roosters is more extensive, involving multiple levels such as redox regulation, central carbon metabolism, amino acid metabolism, and energy metabolism.

### Integrated immunometabolic networks centered on CCR2

**Fig. 6:**
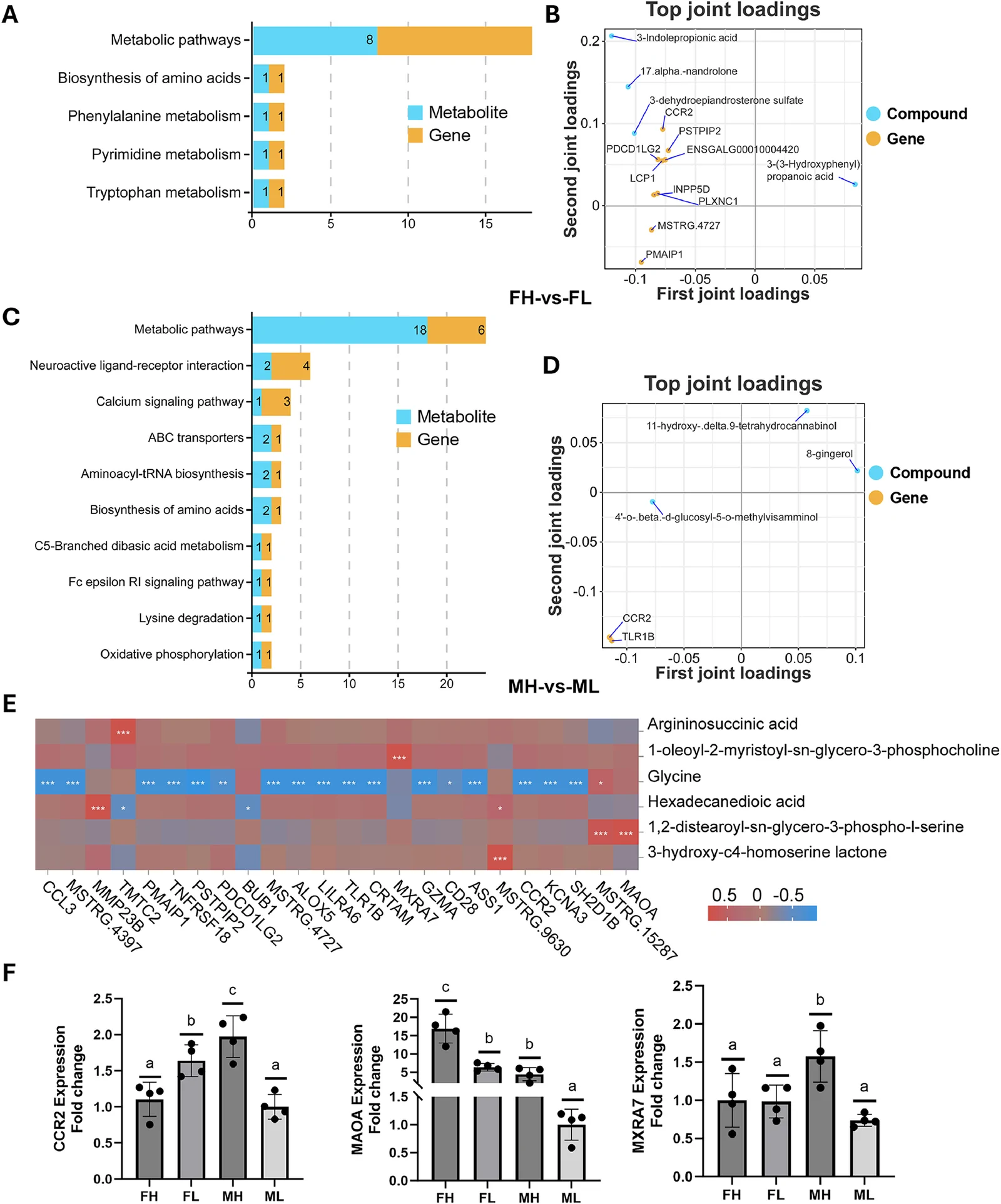
Integrated transcriptomic and metabolomic analyses identify key immune-metabolic hubs associated with H/L ratio. (A) Shared pathways identified by integrated analysis in hens. (B) Top joint loadings from the integrated model in hens, showing representative genes and metabolites contributing to group separation. (C) Shared pathways identified by integrated analysis in roosters. (D) Top joint loadings from the integrated model in roosters. (E) Pearson correlation heatmap of representative genes and metabolites. (F) RT-qPCR validation of the relative expression levels of *CCR2, MAOA*, and *MXRA7* in the female high-H/L (FH), female low-H/L (FL), male high-H/L (MH), and male low-H/L (ML) groups. Different lowercase letters indicate significant differences among groups (*P* < 0.05).

Multi-omics integrated analysis further revealed sex-dependent organizational patterns of H/L ratio-related responses. In hens, the shared pathways between transcriptomic and metabolomic datasets primarily included metabolic pathways, biosynthesis of amino acids, phenylalanine metabolism, pyrimidine metabolism, and tryptophan metabolism (Fig. 6A), suggesting a close coupling between transcriptional variation and amino acid- or nucleotide-related metabolic processes. Joint loading analysis showed that several immune- and metabolism-related genes, including *CCR2, PSTPIP2, PDCD1LG2, LCP1, INPP5D*, and *PLXNC1*, were positioned close to metabolites such as 3-indolepropionic acid, 17.alpha.-nandrolone, and 3-dehydroepiandrosterone sulfate in the loading space (Fig. 6B). These results indicate that H/L-associated responses in hens involve a relatively complex immunometabolic correlation network composed of multiple gene and metabolite nodes.

In roosters, the shared pathways were more extensive and included metabolic pathways, neuroactive ligand-receptor interaction, calcium signaling pathway, ABC transporters, and aminoacyl-tRNA biosynthesis (Fig. 6C). However, despite this broader pathway distribution, joint loading analysis showed that the major gene loadings were mainly concentrated on CCR2 and TLR1B, which formed relatively discrete association structures with metabolites putatively annotated as 11‑hydroxy-delta-9-tetrahydrocannabinol, 8-gingerol, and 4′-O-β-d-glucosyl-5-O-methylvisamminol (Fig. 6D). Compared with the more broadly distributed gene–metabolite associations observed in hens, this pattern suggests that the H/L-associated integrated network in roosters is relatively simplified and more centered on a limited number of key gene nodes.

Correlation heatmaps further showed extensive associations between candidate metabolites—including argininosuccinic acid, 1-oleoyl-2-myristoyl-sn‑glycero-3-phosphocholine, glycine, hexadecanedioic acid, 1,2-distearoyl-sn‑glycero-3-phospho-l‑serine, and 3‑hydroxy-c4-homoserine lactone—and genes related to immunity and metabolism (Fig. 6E). In addition, RT-qPCR validation confirmed the differential expression patterns of *CCR2, MAOA*, and *MXRA7* across sex and H/L groups (Fig. 6F), supporting the reliability of the key nodes identified by the integrated analysis. Overall, these results suggest that CCR2 may represent a shared candidate integration node linking H/L-associated immune and metabolic variation in both hens and roosters.

## Discussion

This study characterized the hepatic molecular profiles associated with divergent peripheral blood H/L ratios in Yellow-legged Ma chickens. The H/L ratio is commonly used in poultry as an important hematological indicator for evaluating physiological stress, immune status, and disease resistance-related phenotypes (Thiam et al., 2022a, 2022b). In the present study, individuals with high and low H/L ratios remained relatively stable under basal physiological conditions, with no obvious histopathological lesions in the liver or spleen, no significant changes in serum transaminase activities, and no marked imbalance in hepatic oxidative stress markers. These results indicate that the hepatic transcriptomic and metabolomic differences observed in this study were not accompanied by detectable overt liver injury or oxidative imbalance under the conditions examined. These molecular differences may be associated with interindividual variation in basal hepatic immunometabolic profiles; however, further functional validation is required to determine whether they correspond to differences in immune competence or disease resistance. Therefore, this study provides tissue- and molecular-level information for further evaluating the biological significance and potential utility of the H/L ratio as a phenotypic indicator in poultry health monitoring and disease-resistance-oriented breeding.

Notably, this study revealed a marked sex-associated differences in the liver molecular profiles associated with the H/L ratio, suggesting that the H/L ratio may correspond to distinct immunobiological features in roosters and hens. In hens, low-H/L individuals showed upregulated hepatic expression of genes such as ACOD1, MX1, OASL, and TET2 (Fig. 2D). Among these genes, OASL and MX1 are typical interferon-stimulated genes that are commonly involved in host antiviral responses (Chen et al., 2021; Wang et al., 2024). Our previous study also demonstrated that elevated TET2 expression exerts a significant antiviral effect (Chen et al., 2025). In addition, ACOD1 has been reported to be closely associated with immunometabolism and anti-infective responses (Wu et al., 2023). These findings indicate that, compared with high-H/L hens, low-H/L hens exhibit higher hepatic expression of multiple immune-related molecules, suggesting a potentially more active antiviral or innate immune response state.

By contrast, in roosters, low-H/L individuals showed lower hepatic expression of immune-related genes such as IFNLR1, TRIM39, ACOD1, and TLR4 than high-H/L individuals. Particularly noteworthy, TLR4, ACOD1, and CCR2 displayed sex-inconsistent expression patterns between the high- and low-H/L groups, indicating that the liver immune molecular features associated with the H/L phenotype are markedly influenced by sex. Previous studies have shown that males and females differ systematically in immune responses (Klein and Flanagan, 2016; Sharma et al., 2025; Tetel et al., 2022), and similar sex-related differences have also been observed in birds (Vincze et al., 2022). Moreover, liver immunometabolic characteristics themselves may exhibit sex dependency (Kasarinaite et al., 2023). Therefore, the differentially expressed genes observed in this study cannot be directly equated with an overall enhancement or reduction in immune competence. Nevertheless, they clearly demonstrate that sex and H/L stratification jointly shape the hepatic immune-related transcriptomic landscape. Accordingly, analyses based solely on H/L values without sex stratification may obscure sex-dependent regulatory signals and underestimate the immunobiological differences reflected by the H/L ratio in individuals of different sexes.

Metabolomics is an essential tool to uncover the pathogenic mechanisms underlying chicken diseases (Zheng et al., 2025). Our results showed that the number of differentially abundant hepatic metabolites between high- and low-H/L hens was markedly lower than that observed in roosters (Fig. 4A and B), further indicating that H/L stratification is associated not only with changes in hepatic immune-related transcriptomic profiles but also with systematic divergence in hepatic immunometabolic states. This finding is consistent with the notion that immune responses and metabolic regulation exhibit sex-dependent differences (Dunn et al., 2024; Vincze et al., 2022). Pathway enrichment analysis showed that the metabolic differences between high- and low-H/L hens mainly involved arginine biosynthesis, α-linolenic acid metabolism, and pyrimidine metabolism, pathways that are closely related to immune cell activation, inflammatory mediator production, and immune regulation (Izadi Yazdanabadi et al., 2024; Marti and Reith, 2021) . In addition, metabolites such as 3-indolepropionic acid showed prominent contributions in the integrated analysis, suggesting that gut-derived tryptophan metabolites may participate in maintaining hepatic immune homeostasis in hens with different H/L ratios (Luo et al., 2025). By contrast, the metabolic differences between roosters with different H/L ratios were mainly enriched in pathways related to histidine metabolism and glutathione metabolism, suggesting that the corresponding phenotype in roosters may rely more on the maintenance of redox homeostasis and antioxidant defense (Gasmi et al., 2024; Li et al., 2024; Lin et al., 2024).

Multi-omics integrative analysis further suggested that CCR2 as a potential candidate node linking the H/L phenotype, hepatic immune regulation, and metabolic remodeling. CCR2 encodes a chemokine receptor involved in monocyte/macrophage recruitment, inflammatory amplification, and regulation of the tissue immune microenvironment (Kadomoto et al., 2021; Wu and Ma, 2024). In chickens, CCR2 transcripts have been detected in T-cell populations, and CCR2 expression in CD4-positive cells responds to mitogenic stimulation, supporting a possible role in avian immune-cell activation or trafficking (Annamalai and Selvaraj, 2010). Thus, the CCR2 association observed here may reflect variation in the hepatic immune-cell composition or immune microenvironment; however, the cellular source and causal function of CCR2 were not determined in the present study. In addition, IFNLR1 and ITIH5, identified through sex-stratified transcriptomic screening and supported by RT-qPCR validation, may warrant further evaluationas auxiliary molecular indicators associated with the H/L ratio. IFNLR1 is the ligand-specific receptor subunit of the type III interferon receptor complex. In chickens, IFNLR1 mediates type III interferon signaling and contributes to antiviral responses and immune regulation in chickens in a virus- and tissue-dependent manner (Alhussien et al., 2026). ITIH5 encodes an extracellular-matrix-associated inter-α-trypsin-inhibitor heavy chain implicated in hyaluronan and extracellular-matrix stabilization. Direct functional evidence for ITIH5 in chickens remains limited, although its hepatic expression has been reported to respond to nutritional perturbation in broilers (Sevane et al., 2014). We therefore interpret ITIH5 as an exploratory candidate associated with tissue-microenvironmental or metabolic remodeling rather than as an established avian immune effector.

It should be noted that this study had a limited sample size and focused on a single chicken breed; therefore, the generalizability of these findings needs to be validated in larger populations with diverse genetic backgrounds. In addition, no pathogen challenge experiment was performed in this study, so the relationship between H/L-associated molecular features and actual anti-infective capacity remains to be further confirmed. Future studies should combine H/L ratio stratification with pathogen infection models, longitudinal sampling, independent population validation, and functional experiments on candidate markers such as IFNLR1, ITIH5, and CCR2, to further determine whether these molecular features can be used to select chickens with stronger immunological robustness and disease resistance potential.

## Conclusion

In summary, this study demonstrates that divergence in the peripheral blood H/L ratio of Yellow-legged Ma chickens is closely associated with hepatic transcriptomic and metabolomic characteristics, with clear sex-dependent patterns. Low-H/L hens mainly exhibited immune-related transcriptional activation, whereas H/L-associated differences in roosters were more prominently reflected in metabolic remodeling. Multi-omics integrative analysis suggested that CCR2 may represent a potential candidate node linking H/L-associated immune and metabolic variation, while IFNLR1 and ITIH5 may serve as candidate auxiliary molecular indicators. Overall, this study provides molecular-level support for the use of the H/L ratio as a phenotypic indicator in poultry health monitoring and disease-resistance-oriented breeding.

## Ethics statement

The chickens were housed at Yangzhou Lihua Poultry Breeding Co., Ltd., with ad libitum access to water and a commercial diet. All animal procedures complied with applicable laws and relevant guidelines and were approved by the Animal Ethics Committee of Yangzhou University (permit no. 202508005).

## Data and availability statement

The data supporting the findings of this study are included in the article. The datasets generated and/or analyzed during the current study are available from the corresponding author upon reasonable request.

## Funding support statement

This study was financially supported by the National Key Research and Development Program of China (2025YFF1000100), the China National Broiler Industry Technology System (CARS-41), the Yangzhou University High-level Talents Support Program.

## Author contributions

**Shihao Chen:** Writing - original draft, Formal analysis, Data curation, Conceptualization.**Qiangzhou:** Wang performed key experiments, assessed the consistency and scientific significance of the experimental findings, contributed to the interpretation of the results, and drafted the relevant Methods and Results sections. **Xuan Huang:** contributed to the design of the experimental work, performed key experiments, and drafted the relevant Methods and Results sections. **Qingqing Cai:** analyzed and interpreted the experimental data presented in the figures, performed key experiments, and drafted the corresponding parts of the Results section. **Xiaoxian Ru:** contributed to the analysis and biological interpretation of the experimental findings, integrated the results into the overall conclusions of the study, and drafted the related Results text. **Pengfei Chu:** contributed to the design of the sample-related experimental work and the criteria for sample selection, participated in interpreting the data generated from these samples, and drafted the relevant Methods and Results sections. **Hao Bai:** designed and implemented the computational analysis approach used in the study, conducted the associated data analyses, interpreted the analytical outputs, and drafted the corresponding Methods and Results sections. **Guobin Chang:** contributed to the conception and scientific design of the study, participated in the interpretation of the principal findings and development of the conclusions, and drafted substantive parts of the Introduction.

## Disclosures

The authors have declared that no conflict of interest.
